# Dialogue Between the Clock Gene Bmal1 and Retinopathy: What Is the Exact Relationship?

**DOI:** 10.1111/cns.70490

**Published:** 2025-11-17

**Authors:** Yongheng Cui, Haoyan Li, Wenhui Fan, Hao Wu, Jiale Wang, Chengkang Qu, Ning Pu, Ye Tao

**Affiliations:** ^1^ Department of Ophthalmology, Henan Eye Hospital, Henan Provincial People's Hospital People's Hospital of Zhengzhou University Zhengzhou China; ^2^ College of Medicine Zhengzhou University Zhengzhou China

**Keywords:** circadian rhythms, clock genes, eye diseases, retinal degeneration

## Abstract

**Background:**

Circadian clock coordinates the physiologic and behavioral activities with a 24‐hour solar rhythm to maintain the temporal homeostasis of the body. In the mammalian retina, the circadian system regulates the physiological function of this organ. The realm of ocular circadian rhythm has earned kinds of research interest as the circadian rhythms dysfunction will disrupt the retinal homeostasis. *Bmal1* functions as a major transcriptional regulator of the circadian clock.

**Results:**

In the retina, *Bmal1* mediates the processing of light information, sustains photoreceptor viability and governs neurotransmitter release. Moreover, *Bmal1* gene is believed to be a pathologic cofactor of the diabetic retinopathy (DR), age‐related macular degeneration (AMD), premature aging and refractive myopia. To date, the precise mechanisms underlying the pathological effects mediated by *Bmal1* remain incompletely elucidated.

**Conclusions:**

This review presents recent findings and evidence regarding the contributory role of *Bmal1* in retinal degeneration and its deficits, while exploring its therapeutic potential. And th review provides a comprehensive analysis of the underlying mechanisms of the clock gene *Bmal1* in other diseases, with the aim of offering insights into innovative therapeutic strategies for retinopathy.

## Graphical abstract

Circadian rhythm is offering novel insights into the pathogenesis of retinopathy. *Bmal1*, a key transcriptional regulator of the circadian clock, serves as a critical pathologic cofactor in retinal degeneration. Deficiency in *Bmal1* contributes to a broad spectrum of retinal pathological alterations, and modulating the clock gene *Bmal1* could potentially inform innovative treatment strategies for retinal diseases.

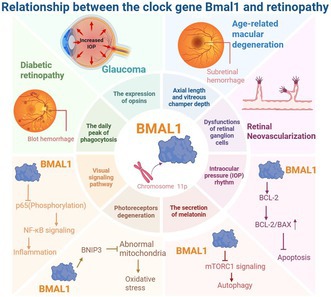

## 1 Introduction

Circadian clock is an internal timekeeping mechanism that drives the daily oscillations of various physiologic and behavioral processes within a 24‐h period [1]. The accomplishment of this process is achieved through a series of intricate gene expression programs. In mammals, the circadian clock system is a hierarchy consisting of numerous oscillators at the organismal, cellular, and molecular levels. At the organismal level, the suprachiasmatic nucleus (SCN) is the central pacemaker that integrates inputs of light information and ultimately regulates the clock gene expression and physiological rhythms. At the cellular level, the SCN is composed of various oscillatory neurons that are ultimately coupled into a circadian unit [2, 3]. Typically, circadian rhythms and clock genes interplay with each other, thereby forming a complex network. In the mammalian retina, there exists an autonomous circadian clock system that functions independently of the SCN. Retinal rhythms combine with light/dark adaptive processes to modulate the physiology of the retina. The wave forms of the electroretinogram (ERG), light responses of cones, visual sensitivity, dopamine production, and axial length variation all manifest typical circadian rhythms [4]. It is polyphyletic since the circadian genes are expressed in different retinal cell populations [5, 6]. *Bmal1* is a core clock gene that plays a critical role in regulating circadian rhythms, and its dysregulation has been linked to various pathophysiological processes of retinal diseases. For example, studies have shown that *Bmal1* modulation can affect photoreceptor survival and function, indicating a potential connection between circadian rhythms and photoreceptor degeneration [7].

**FIGURE 1:**
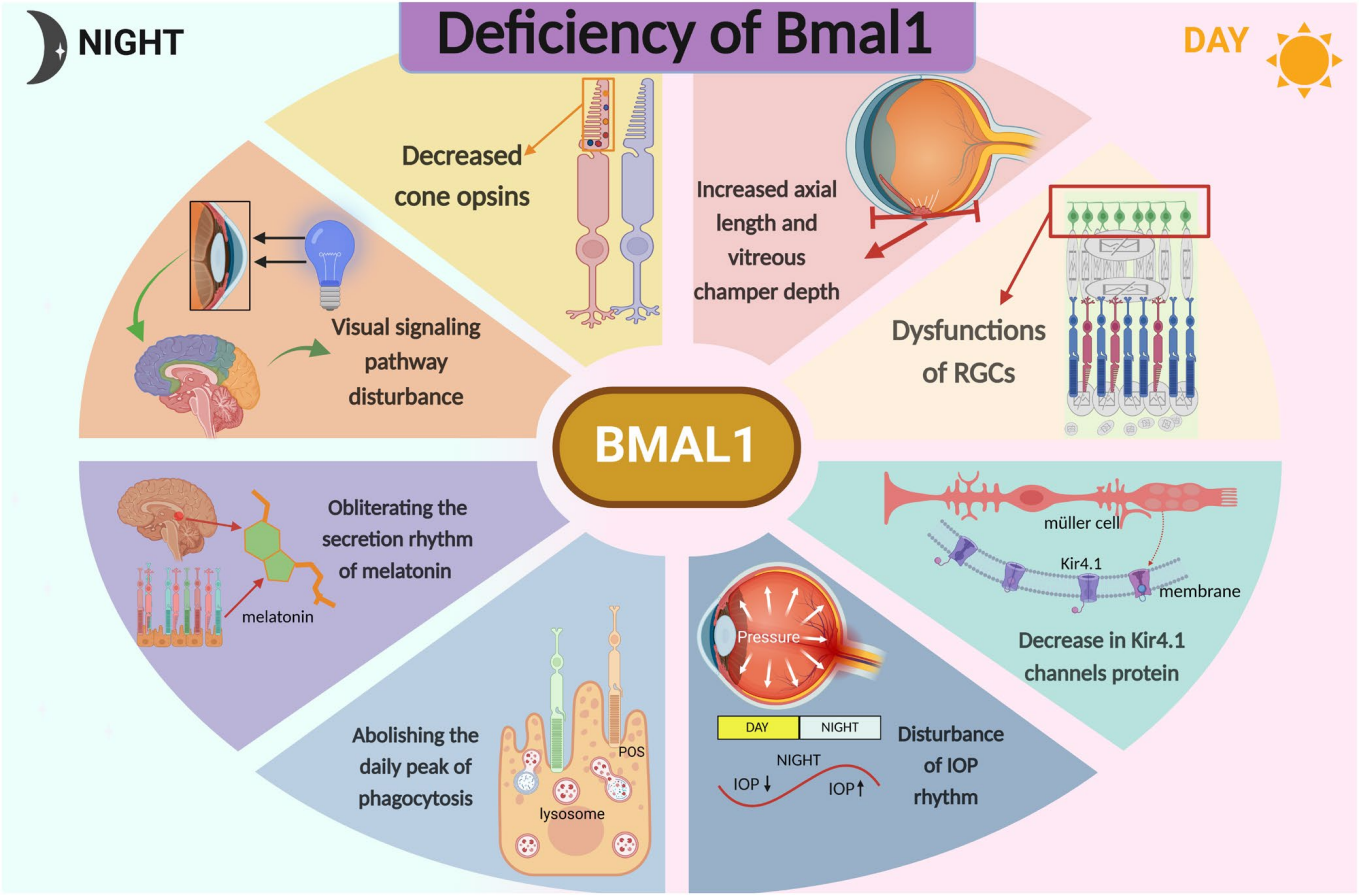
An overview of the basic functions of *Bmal1*. *Bmal1* plays a crucial role in circadian rhythm, encompassing vision/image processing, photoreceptor viability, the circadian secretion rhythm of dopamine and melatonin, IOP regulation, RGCs function, decreased Kir4.1 protein levels, and reduced cone opsin expression.

Recent studies on multiple *Bmal1* deficiency phenotypes reinforce the notion that *Bmal1* deletion or conditional knockdown can cause circadian rhythm‐related disorders, such as the disruption of intraocular pressure (IOP) rhythm, reduced opsin expression, and the abolition of melatonin secretion rhythm (Figure 1). Additionally, dysregulation of *Bmal1* has been detected in retinopathies such as age‐related macular degeneration (AMD) and diabetic retinopathy (DR), in which altered circadian rhythms may disturb the retinal pathophysiological process [8, 9]. Furthermore, several pioneering studies have explored the interactions between the immune microenvironment, the gut microbiome, and circadian rhythms, thereby envisioning the broader implications of circadian rhythms on retinopathies [10, 11, 12]. The mounting body of evidence indicates that *Bmal1* serves as a bridging factor between circadian rhythms and retinal diseases, providing novel insights into the development of circadian‐based therapeutic strategies. It has been demonstrated that *Bmal1* can affect many crucial factors for retinal development, including neurotransmitter release, dopamine synthesis, intraocular pressure fluctuations, and photoreceptor photosensitivity.

The term “clock genes” is also used to refer to “circadian clock genes.” These genes form the molecular foundation for rhythm expression [13]. Brain and muscle ARNT‐like protein 1 (BMAL1) is a core gene that plays an essential role in maintaining circadian rhythms. It is expressed in both the SCN and peripheral tissues. The CLOCK (Circadian Locomotor Output Cycles Kaput) gene forms heterodimers with BMAL1, which subsequently bind to E‐box elements and activate the transcription of clock‐controlled genes (CCGs) [14, 15]. The PER and Cry proteins are considered to be primary clock genes, and it has been demonstrated that their transcription is activated by the BMAL1/CLOCK heterodimer. Moreover, the PER and CRY proteins have been shown to inhibit the transcription of *Bmal1* and *Clock*, thereby establishing a negative feedback loop [16, 17]. Additionally, the nuclear transcription factor retinoid acid receptor‐related orphan receptor α (RORα) has been shown to enhance *Bmal1* transcription, while the nuclear receptor Rev‐erbα suppresses it [18].

Collectively, these circadian genes and encoded proteins form an integrated web to modulate physiologic rhythms. Circadian clock located in the retina is initially discovered in mammals' extra‐SCN circadian oscillator [19]. In the retina, the clock gene *Bmal1* is expressed in various cells, including the retinal pigmented epithelium (RPE), the inner nuclear layer (INL), the ganglion cell layer (GCL), and the cone photoreceptors [6, 19, 20, 21] (Table 1). Furthermore, it has been demonstrated that *Bmal1* can affect many crucial factors for retinal development, such as the release of neurotransmitters, dopamine synthesis, fluctuations of intraocular pressure, and photoreceptor photosensitivity. In this context, *Bmal1* may function as an integral component of the circadian rhythms. Herein, we introduce recent advances in circadian rhythms, with a particular focus on the role of *Bmal1* in the physiopathology of retinopathy. We further summarize the potential therapeutic strategies targeting the *Bmal1* and delve into the future perspectives and limitations of circadian clock gene therapy for retinopathy. These findings could enrich our knowledge of circadian gene and provide a valuable reference for clinical translation.

**TABLE 1:** *Bmal1* expresses in various types of retinal cells and its potential role.

Retinal cells	The expression of *Bmal1*	The effect of Bmal1 deficiency	The function of cell	Method of *Bmal1* deletion	References
Retinal ganglion cells	+	Causes a drastic reduction in neovascularization	Related to retinal angiogenesis	Chx10Cre	[9]
Rod cells	+	Subjective day/night difference in dark‐adapted ERG is lost	It expresses retinal rhodopsin visual pigments and is involved in the regulation of visual functions	CRE recombinase	[22]
Cone cells	+	A decrease in M opsin‐expressing cones and a concurrent increase in the density of dual cones	*Bmal1* is required for the maintenance of S opsin gradient and regulates the expression of M opsin	Using cone‐specific HRGP‐Cre transgene to delete *Bmal1*	[23]
Horizontal cells	+	—	—	—	[24]
Astrocytes and Müller cells (glial cells)	+	Decreases the b‐wave amplitude under light‐adapted conditions	As part of the brain's circadian circuit to regulate daily behaviors	Crossing a knock‐in mouse line bearing a Tamoxifen (TM)‐inducible CreERT2 recombinase	[25]
RPE	+	Reduces the daily peak of phagocytic activity by the RPE	Controls the diurnal peak in phagocytosis of photoreceptor outer segments (POS)	CRE recombinase	[26]
Astrocytes	+	Impairs the molecular clock in the hypothalamus, and alter circadian motor behavior, cognition, and lifespan Affects metabolic balance and glucose homeostasis	Astrocytes critically modulate hypothalamic neural circuits controlling energy homeostasis	Glial Glu and aspartate transporter (Glast‐Cre)	[27]

## 2 Bmal1 and Molecular Clockwork

BMAL1 protein is a basic helix–loop–helix (bHLH)/PAS (Period‐Arnt‐Single‐minded) domain transcription factor in the transcription‐translation feedback loop (TTFL). Notably, the deletion of *Bmal1* can result in complete ablation of circadian rhythms in mammals [15]. BMAL1 protein interacts with chromatin‐modifying complexes to acetylate histones and rapidly forms CLOCK:BMAL1 heterodimers with it heterodimeric partner CLOCK [28]. The bHLH/PAS CLOCK protein also contributes to the circadian oscillator. Subsequently, the sumoylation, phosphorylation, and acetylation modulate CLOCK:BMAL1 transcriptional activity [29, 30]. The CLOCK:BMAL1 heterodimer binds to DNA via hydrogen bonds between serine residues, and DNA at the recognized E‐box site can directly regulate transcription of core circadian clock genes. It is well known that the E‐box sites are located approximately 20 base pairs upstream of genes and have a major “5′‐CACGTG‐3′” or “5′‐CACGTT‐3′” canonical motif [31]. Circadian rhythms are governed by oscillating circadian molecular clocks via molecular feedback loops. The core oscillator consists of at least three mutually interlocking feedback loops including a transcriptional negative‐feedback loop and two accessory feedback loops [32]. The CLOCK:BMAL1 heterodimer acts as a core factor to activate and drive these feedback loops.

**FIGURE 2:**
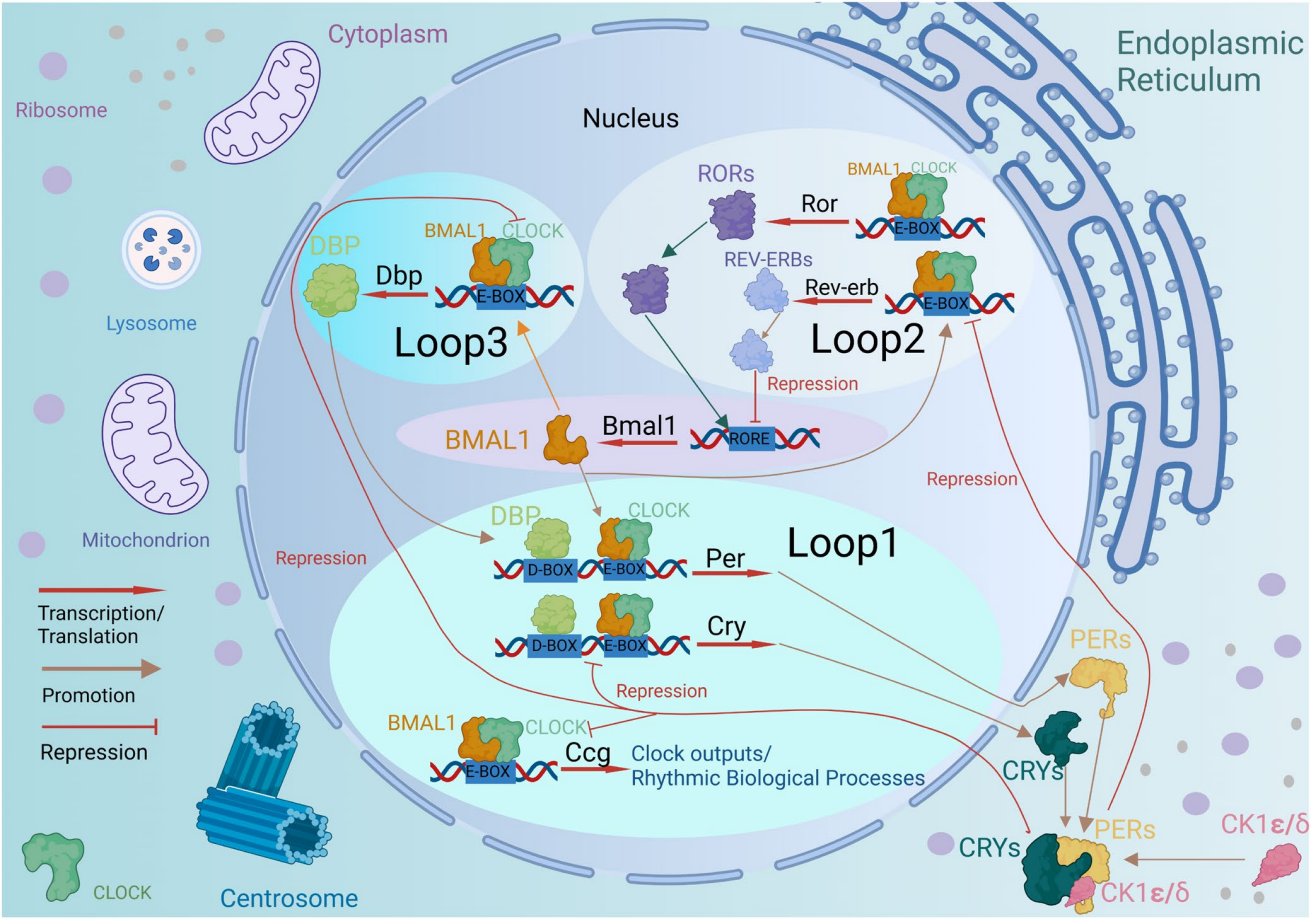
Circadian gene network in mammals. The heterodimer formed by BMAL1 and CLOCK binds to the E‐box present on the promoter region of *Per* and *Cry* genes, leading to the inhibition of their own transcription by PER and CRY proteins. In a second feedback loop, CLOCK/BMAL1 complex transactivates *Rev‐erbα*, *Rev‐erbβ*, and *Rora* genes. Subsequently, REV‐ERBα, REV‐ERBβ, and RORA proteins compete for binding to RRE elements in the *Bmal1* promoter region, thereby driving a daily rhythm of *Bmal1* transcription. These interconnected transcriptional feedback loops represent the three major regulators governing most cycling genes' expression. Different combinations of these factors generate distinct phases of transcriptional rhythms.

The first accessory feedback loop consists of *Rors (α/β/γ)* and *Rev‐erb (α/β)*. The CLOCK:BMAL1 heterodimer can activate the expression of retinoic acid receptor‐related orphan receptors (RORs) and REV‐ERB (α/β) via binding to the E‐box [33, 34]. However, they in turn translocate back to the nucleus to compete for receptor‐related orphan receptor response elements (ROREs) in the promoter of *Bmal1* [33, 35]. RORs play a role in the *Bmal1* activation and conversely, REV‐ERB (α/β) can inhibit the expression of *Bmal1* [36, 37]. The PAR bZIP transcription factor family includes activators such as D‐Box Binding PAR BZIP transcription factor (DBP) and hepatic leukemia factor (HIF), which form the second accessory feedback loop. DBP is regulated by the CLOCK:BMAL1 via its E‐box and can act through D‐box elements in target genes including *Per* and *Cry* [30]. These three interlocking transcriptional feedback loops form the foundation of the molecular oscillator, thereby governing the circadian network of clock‐controlled genes (Figure 2).

## 3 Crosstalk Between Bmal1 and Retinal Physiology

The retina is composed of a diverse array of specialized cells that are integral to its structure and function. These cells collectively perform essential functions, and retinal diseases often arise due to disruptions in the specialized cells. Photoreceptors (rods and cones) are responsible for light transduction [38]. Disruptions of phototransduction in their outer segment maintenance would result in progressive vision loss. Oxidative stress in RPE cells, which is essential for the removal of photoreceptor debris and the regulation of the visual cycle, has been shown to trigger the formation of lipid‐rich deposits (drusen) which in turn exacerbate photoreceptor degeneration [39, 40]. Congenital stationary night blindness (CSNB) is a term used to describe a group of genetically determined conditions that result in impaired vision during the nighttime hours. Mutations causing CSNB have been shown to localize to the dendritic tips of ON‐bipolar cells, encoding other proteins with a role in postsynaptic signal transmission [41, 42]. Retinal horizontal cells (HCs) are interneurons that play a crucial role in visual processing by modulating photoreceptor output and enhancing spatial and chromatic contrast [43]. Although not directly investigated, HC dysfunction may indirectly contribute to DR. Diabetic hyperglycemia disrupts retinal calcium homeostasis, potentially impairing HC‐mediated lateral inhibition and thereby exacerbating vascular leakage or neural degeneration. Retinal ganglion cells (RGCs), which function as the retina's output neurons, exhibit a high degree of susceptibility to elevated intraocular pressure in the context of glaucoma [44]. The failure of axonal transport, in conjunction with the onset of neuroinflammation, contributes to the process of cellular apoptosis loss [45]. Notably, Müller glia, the retina's primary support cells, contribute to the pathophysiological process of DR by undergoing reactive gliosis, thus disrupting potassium/water homeostasis and promoting vascular leakage [46, 47]. Recent research has confirmed the involvement of microglia in the development of various retinopathies, such as AMD, DR, and glaucoma. This participation is characterized by the expression of numerous inflammatory markers, the release of proinflammatory cytokines (such as TNF‐α, IL‐1β, MCP‐1), and the subsequent amplification of inflammatory cascade responses following activation [48, 49, 50]. The pathophysiological mechanisms that underpin retinal disease highlight the necessity to perform multifaceted research.

### 3.1 Role of Bmal1 in Photoreceptors

Photoreceptors, comprising cone and rod cells, are specialized sensory neurons in the retina which are responsible for converting light stimuli into nerve impulses [51]. Rod cells exhibit higher sensitivity to low light conditions, facilitating night vision, whereas cone cells excel in color discrimination and visual perception during daylight [52]. The functional differences between rods and cones are caused by the varied expressions of specific light‐sensing opsins that regulate the key signaling pathways of visual function [53, 54]. Rod cells only express one opsin, known as rhodopsin, while cone cells possess a variety of opsins and are classified based on their spectral sensitivity. The mammalian retina displays a wide array of daily rhythms in biochemical and cellular processes, allowing visual function to adapt to the light/dark (LD) cycle [38]. The rhythmic processes encompass the mRNA expression of photopigments and genes related to phototransduction in both rods and cones [55]. Additionally, targeted deletion of *Bmal1* specifically in the retina, similar to the knockout of *Bmal1* in the entire organism, leads to a reduction in both scotopic and ERG b‐wave amplitudes. Therefore, disruption of *Bmal1* is likely to impact the transmission of light information from rods and cones to bipolar cells and/or its processing in the inner retina. The researchers found that at 26 months, Chx10Cre; Bmal1^fl/fl^ mice had about a 60% reduction in the number of cone outer segments and cone nuclei compared to younger mice [56]. Specifically, in the cone pathway, significant effects include diminished viability of cones, shortened lengths of OS and IS, as well as disruption in both structure and function of cones. Moreover, these features would be accentuated in aging mice. In the rod pathway, the dendritic processes of rod bipolar cells were notably stunted at both ages, while the dendrites of cone bipolar cells appeared to develop normally. These findings indicate that the effects of *Bmal1* deletion on the rod and cone visual pathways are likely mediated by distinct mechanisms. Researchers also specifically demonstrated the significance of *Bmal1* in the light‐adapted ERG, wherein the b‐wave exhibits greater amplitude during the subjective day compared to the subjective night [57]. Interestingly, a study observed diurnal variations in ERG a‐ and b‐waves in control mice carrying one allele of *Bmal1* specifically in rods, with higher amplitudes during the subjective night. Together, these data demonstrated the fundamental role of retinal clocks in the structural integrity and functional performance of retinal tissue. However, the molecular pathways that link cell specific clocks to retinal health and function remain to be elucidated. Recently, researchers knocked out *Bmal1* in the rod photoreceptors of the P23H mutant mouse model (P23H Rho) [7]. The results reveal that conditional, rod‐specific KO of *Bmal1* markedly exacerbates the retinal RP phenotype in P23H mice. Compared with P23H Rho mutant mice, double mutant mice display a significantly greater number of differentially expressed genes in the retina, involving pathways such as neurogenesis, phototransduction cascades, and metabolic processes. This not only elucidates a connection between circadian clock dysfunction and retinal degeneration but also potentially promises an avenue for the restoration of vision in retinal degeneration through the targeted manipulation of the clock gene *Bmal1*.

Recently, the clock gene *Bmal1* that controls thyroid hormone‐mediated spectrum recognition and cone cell photosensitivity is studied [23]. Thyroid hormone (TH) signaling exerts regulatory control over numerous physiological functions, encompassing the modulation of dorso‐ventral cone gradient distribution and cone apoptosis [58, 59]. The study demonstrates that disruption of the circadian clock gene *Bmal1* leads to a perturbation of the dorso‐ventral gradient of S and M opsins, resulting in the upregulation of S opsin expression across the entire retina. Additionally, the transcript levels encoding the thyroid‐activating enzyme Dio2 exhibit oscillations within the retina throughout the circadian day, and it is noteworthy that the circadian clock protein BMAL1 binds to the promoter region of Dio2, thereby providing compelling evidence for a persistent involvement of thyroid hormone signaling in maintaining cone photoreceptors [23]. *Bmal1* also has a close connection with Complex proteins (Cplxs), which function as regulators of the soluble N‐ethylmaleimide‐sensitive factor attachment protein receptors (SNARE) complex and govern the velocity and calcium ion sensitivity of SNARE‐mediated synaptic vesicle fusion [60]. All four Cplx isoforms have been identified in the mouse retina, with Cplx3 and Cplx4 being localized at the synapses of cone photoreceptor ribbons that exhibit relatively lower expression in rods [61]. Depletion of *Bmal1* in cone cells leads to decreased levels of Cplx3 in both cones and rods [62]. In cone‐specific *Bmal1* knockout mice, Cplx3 expression decreases during the day, while Cplx4 expression in the outer plexiform layer remains unaffected after *Bmal1* knockout. Furthermore, a study identifies three E‐boxes within the promoter region of Cplx3, one of which serves as a binding site for CLOCK:BMAL1 complex to control Cplx3 expression [63]. Thereby, the straightforward explanation is that Cplx3 transcription is enhanced through the circadian clock via CLOCK:BMAL1 binding to these E‐boxes. In conclusion, Cplx3/4 acts as a regulator in the adaptation‐dependent availability of synaptic vesicles, especially at photoreceptor ribbon synapses. Given the essential role of *Bmal1* in maintaining cone functionality, it is imperative to develop future therapeutic strategies targeting these genes to restore retinal circuit function; however, further experimental evidence is required to validate this hypothesis.

### 3.2 Role of Bmal1 in Melatonin Synthesis

**FIGURE 3:**
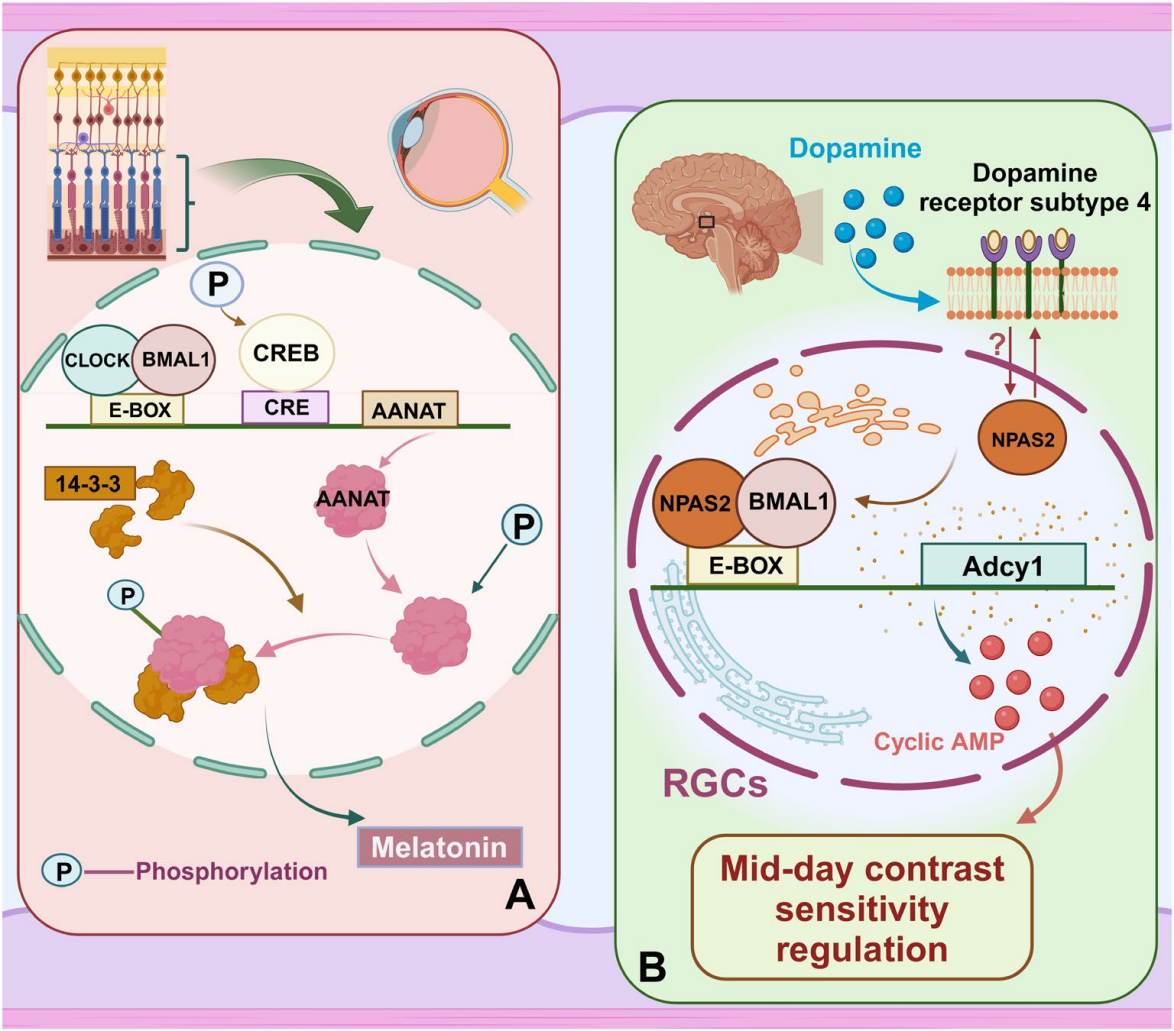
(A) Regulation of retinal melatonin levels by the circadian clock; (B) A model for mid‐day contrast sensitivity regulation. The modulation of contrast sensitivity in the circadian rhythm seems to be mediated by a D4R ⇒ NPAS2 ⇒ cAMP signaling cascade within a specific population of RGCs.

Melatonin is a hormone that is physiologically synthesized in the pineal gland and retina via a well‐defined biosynthetic pathway [64]. In the retina, melatonin production follows a circadian rhythm and predominantly occurs in the photoreceptor layer (Figure 3A). To date, multiple studies have substantiated the existence of a circadian rhythm in retinal melatonin synthesis [65]. Melatonin functions as an exceptional free radical scavenger in nature, playing a pivotal role in regulating the human sleep–wake cycle and modulating inflammatory responses [66]. Melatonin exerts its effects through binding to MT1 (melatonin receptor type 1) and MT2 (melatonin receptor type 2), which are members of the G protein‐coupled Euphorbia receptor family [67]. In rats, MT1 receptors are found in retinal horizontal cells, inner plexiform layer, photoreceptors, and RGCs [68]. The synthesis of melatonin involves a multi‐step pathway with the participation of numerous enzymes, starting from tryptophan. The sequential order of these participating enzymes includes tryptophan hydroxylase (TPOH), aromatic amino acid decarboxylase (AAAD), arylalkylamine‐N‐acetyltransferase (AA‐NAT), and hydroxyindole‐O‐methyltransferase (HIOMT, also known as acetylserotonin‐O‐methyltransferase [ASMT]) [69]. A significant portion of the research on melatonin synthesis regulation has primarily focused on elucidating the regulatory mechanism of AA‐NAT due to its substantial circadian variations in activity, which give rise to an ∼10‐fold melatonin rhythm within the pineal gland [64]. Research has shown that the activity of CLOCK:BMAL1 is dynamically consistent with increased *AANAT* mRNA transcription in mice [70]. Cyclic adenosine monophosphate (cAMP) stimulates *AANAT* gene expression by activating protein kinase A (PKA) and phosphorylating the cAMP‐responsive element‐binding protein (CREB), allowing it to interact with the cAMP response element (CRE) on the *AANAT* gene promoter [71]. Additionally, the elevation of cAMP and PKA activity leads to phosphorylation of AANAT, facilitating its binding to the corresponding protein [72]. Within the retina, the promoter region of the *AANAT* gene contains an E‐box sequence that serves as a binding site for the CLOCK:BMAL1 heterodimer, regulating the rhythmicity of AANAT enzyme [73]. CLOCK:BMAL1 rhythmically controls the expression of type 1 adenylyl cyclase (AC1) mRNA through an E‐box in its promoter region to govern cAMP synthesis [74]. Melatonin synthesis is exclusively regulated in darkness under multiple regulatory mechanisms. Conversely, melatonin can also modulate the *Bmal1* expression. Several studies have shown that the scotopic ERG in melatonin‐deficient mice (C57/Bl6 mice) is not regulated by the circadian clock; this lack of circadian regulation may be attributed to the absence of melatonin signaling [75]. Removal of melatonin signaling affects the *Bmal1* expression and other clock genes in the GCL. A study utilizing melatonin receptor knockout mice shows that melatonin signaling can influence the circadian rhythm of Per1 and Cry2 in the inner retina through MT1 receptors, as well as these same genes in RGCs layer via both MT1 and MT2 receptors; it also affects PER1 and CRY2 protein levels through post‐transcriptional mechanisms rather than mRNA expression [76]. On the basis of our current understanding, it is plausible that melatonin exerts both dependent and independent effects on the clock gene *Bmal1* to modulate retinal physiology.

### 3.3 Role of Circadian Rhythm in the Regulation of Dopamine Release

Dopamine (DA) is a pivotal neurotransmitter involved in numerous physiological functions, encompassing motor control, modulation of affective and emotional states, reward mechanisms, reinforcement of behavior, selected higher cognitive functions, and mid‐day contrast sensitivity (Figure 3B) [77, 78]. It has been demonstrated that dopamine release and synthesis follow a circadian rhythm, with both dopamine levels and its primary retinal metabolite 3,4‐dihydroxyphenylacetic acid (DOPAC) being higher under light conditions than under dark conditions [79, 80]. Dopamine regulates intracellular changes through two metabolic dopamine receptor (DR) families that are G protein‐coupled receptors. Activation of D1 receptors (including D1R and D5R) stimulates cAMP production and protein kinase A activity. Conversely, activation of D2 receptors (including D2R, D3R, and D4R) exerts an inhibitory effect on cAMP production and protein kinase A activity [81]. A previous study demonstrated that the nuclear staining intensity of dopamine cells in the retina of *Bmal1* ^−/−^ mice was significantly reduced compared to wild‐type controls, suggesting the presence of BMAL1 protein expression in dopamine cells [82]. Moreover, activation of D2R‐dependent signaling stimulates the MAPK transduction cascade and enhances recruitment of CREB Binding Protein (CBP) to CLOCK:BMAL1 complex, thereby promoting CBP phosphorylation. Consequently, D2R‐mediated signaling potentiates CLOCK:BMAL1‐driven circadian regulation. Notably, both CLOCK:BMAL1‐dependent activity and light‐induced *mPer1* gene transcription are significantly attenuated in retinas lacking D2R [80]. Therefore, dopamine could potentially serve as an intermediary for transmitting signals from the central to peripheral circadian clock within the retina.

The retinal circadian clock exerts an indirect influence on dopamine levels in the retina through modulating the hormone melatonin. Arguably, one of the extensively investigated effects of melatonin in the vertebrate retina pertains to its modulation of dopamine release, with melatonin acutely inhibiting this process across all vertebrate species [83]. For instance, the circadian rhythm of dopamine release in the mouse retina is likely dependent on MT2 receptors, as it persists even in MT1 knockout animals [75]. The melatonin/dopamine system has been extensively investigated as one of the most prominent clock pathways in the vertebrate retina. In Xenopus and fish, the melatonin release rate is high during the night, while the dopamine release rate is high during the subjective day [84]. Endogenous activation of melatonin receptors at night inhibits dopamine release, supporting the view that the circadian melatonin generates the rhythm of dopamine release. However, the exact mechanism/circuit through which melatonin inhibits dopamine remains unclear. The clock genes are believed to account for the clock‐related effects of melatonin and dopamine on retinal physiology. Future investigations into the role of the clock gene *Bmal1* in regulating the circadian release patterns of melatonin and dopamine may hold great potential for advancing our understandings of retinal physiology.

## 4 Crosstalk Between Bmal1 and Diabetic Retinopathy

DR is a vascular and neurodegenerative disease that arises as a complication of advanced diabetes mellitus. It represents a major complication of chronic hyperglycemia and one of the leading causes of blindness in developed countries. The projections for 2045 are staggering; more than 700 million people worldwide are estimated to have diabetes [85]. The hallmark features of DR mainly include neuroretinal degeneration and vascular injuries. In clinical practice, DR is typically categorized into two primary types: nonproliferative diabetic retinopathy (NPDR) and proliferative diabetic retinopathy (PDR). NPDR is characterized by the presence of microaneurysms, intraretinal hemorrhages, hard exudates, and cotton‐wool spots. Conversely, PDR is distinguished by neovascularization on the retina, preretinal hemorrhage, vitreous hemorrhage, fibrous tissue proliferation, and traction‐related retinal detachment [86]. At the early stages of DR, patients always remain asymptomatic. Significant vision loss usually becomes evident only when the disease progresses to PDR or diabetic macular edema (DME) [87]. Neuroretinal degeneration involves multiple cell types, including glial cells (astrocytes and Müller cells), ganglion cells, endothelial cells, pericytes, and retinal pigment epithelial cells [88]. In DR pathogenesis, the disruption of *Bmal1* expression disrupts retinal homeostasis and accelerates disease progression. Specifically, *Bmal1* has been recognized as a pivotal regulator of Müller cell function and retinal neovascularization [46, 47, 89]. These findings highlight its critical role in preventing the advancement of DR.

### 4.1 Bmal1 Regulates K^+^ Channels in Db/Db Mice Indirectly

The clock gene *Bmal1* exhibits a correlation with insulin receptor substrate‐1 (IRS‐1) and the K^+^ channel (Kir4.1), in regulating pathological changes within DR mice. Various ion channels, ligand receptors, and transmembrane transporter molecules are presented on the membrane of Müller cells, including IRS‐1. DR downregulates the expression of Kir4.1 protein while altering K^+^ conductance, resulting in Müller cells swelling and dysfunction [46, 47]. The diurnal rhythm of Kir4.1 in Müller cells has been previously reported, and it has been demonstrated that BMAL1 directly modulates this rhythm, potentially through its interaction with E‐box elements [47]. Moreover, the functionality of Kir4.1 channels in Müller cells relies on insulin signaling mediated by IRS‐1, and the diurnal rhythm of Kir4.1 is synchronized with that of IRS‐1. Researchers have demonstrated that *Bmal1* maintains the intrinsic rhythm of insulin secretion, while IRS‐1 potentially modulates Kir4.1 channels via the circadian clock gene *Bmal1*, thereby implicating their significant involvement in Müller cell dysfunction [47, 90]. Furthermore, silencing of IRS‐1 in rat Müller cells (rMC‐1) results in a reduction in *Bmal1* mRNA, BMAL1 protein, and Kir4.1 protein levels. Additionally, rMC‐1 cells administered with siRNA targeting the clock gene *Bmal1* exhibits decreased expression of Kir4.1 [90]. Additionally, the circadian regulatory protein BMAL1 plays a crucial role in connecting IRS‐1 and Kir4.1, thereby revealing a novel mechanism underlying the decrease of Kir4.1 in diabetes and providing new insights into the involvement of circadian dysrhythmia in Müller cell dysfunction observed in diabetes.

### 4.2 Bmal1 Indirectly Affects the NF‐κB Signaling Pathway

NF‐κB is a family of transcription factors that are of central significance in several immunity processes, including development, cell growth and survival [91]. Experimental models have shown that there exists an inflammatory environment in the DR retina with the upregulation of IL‐6 and TNF‐α, and *Bmal1* can indirectly amplify the NF‐κB signaling pathway in mice with DR [92]. Studies have demonstrated that *Bmal1* deficiency results in CLOCK release, promoting p65 phosphorylation to amplify the NF‐κB signaling pathway [93]. Notably, overexpression of *Bmal1* could enhance CBP levels to activate the NF‐κB pathway, which subsequently inducing inflammatory cytokine release through enhancing p65 activity. The downstream target genes regulated by activated NF‐κB pathway further facilitate the invasion and metastasis in breast cancer cells [94]. Intriguingly, the pro‐inflammatory transcription factor NF‐κB subunit RELA, like the clock repressor CRY1, represses the transcriptional activity of CLOCK:BMAL1 on the cis‐element of the circadian E‐box [95]. NF‐κB exerts a remodeling effect on the BMAL1/CLOCK‐E‐box complex. Further investigations are warranted to elucidate the coordinated regulation of E‐box transcription by BMAL1, CLOCK, and NF‐κB.

### 4.3 Role of Bmal1 Between Melatonin and DR

Melatonin (N‐acetyl‐5‐methoxytryptamine), primarily synthesized by the pineal gland, is also endogenously produced in the retina of various vertebrate species. AANAT serves as the principal regulatory enzyme in melatonin synthesis, exhibiting a pronounced circadian variation in both its mRNA expression level and enzymatic activity within the rat retina. Recently, there has been significant attention focused on melatonin and DR. Melatonin plays a pivotal role in the pathogenesis of DR, encompassing the maintenance of iBRB, modulation of autophagy, regulation of inflammatory responses, and attenuation of oxidative stress [96]. It has been demonstrated that a significant decrease in pineal melatonin synthesis is observed in diabetic rats induced by alloxan or STZ (Amaral FG, personal communication, 2010), as well as in hamsters [97]. Interestingly, STZ‐induced diabetic rats exhibited disrupted rhythmicity in *AANAT* activity, thereby providing a highly plausible hypothesis that the reduction in melatonin synthesis is attributed to perturbations in the circadian regulation of the AANAT enzyme. Moreover, the involvement of the CLOCK:BMAL1 complex in the regulation of *AC1* and *AANAT* gene expression, along with the crucial role of the cAMP signaling pathway in maintaining *AANAT* stability, is well established. In the diabetic retinas, the diurnal variation in *Bmal1* expression is abolished compared to that observed in the control retinas [65]. It is plausible to hypothesize that the downregulation of *Bmal1* expression may potentially impede AC1 levels, leading to the observed decline in cAMP concentration and subsequent reduction in AANAT activity. Additionally, the flattened expression of *Bmal1* may exert an influence on *AANAT* gene expression. This further emphasizes the crucial importance of investigating the intricate correlation among *Bmal1*, melatonin, and DR. These explorations mark an epoch‐making milestone in clock gene research and cast unprecedented insights into comprehending the retinal circadian rhythms in DR.

### 4.4 Role of Bmal1 and Retinal Neovascularization

Endothelial dysfunction with reduced vascularity is the central pathology of DR vascular disease, which is largely mediated by bone marrow progenitor cells (BMPCs) expressing the *Bmal1* gene [98, 99]. Current research indicates that mice lacking *Bmal1* in their endothelial cells exhibit exacerbated retinal damage following I/R injury compared with WT mice, accompanied by an accelerated increase in the folding of cellular capillaries. The deletion of endothelial BMAL1 orchestrates the progression of microvascular injury, and the absence of *Bmal1* in BMPCs further exacerbates endothelial dysfunction due to their inadequate participation in vascular repair [98]. According to a previous research, the *Bmal1* gene in RGCs owns a robust correlation with retinal neovascularization [89]. The intense diurnal expression of *Bmal1* in the mouse embryo retina is stable after exposure to light stimulus. Retinal neurons can influence the growth of retinal blood vessels in part through light‐mediated signaling pathways, and deletion of *Bmal1* and *Per2* from retinal neurons leads to defects in neovascularization [53, 100, 101, 102]. The deficiency of *Bmal1* and *Per2* in embryonic mouse retinas results in impaired angiogenic function [9, 56, 103]. Specific knockout of *Bmal1* in endothelial cells would exacerbate retinal microvascular damage and induce pathology closely resembling DR. Moreover, systemic deletion of *Bmal1* can promote the production of superoxide radicals in mouse retina [98]. Under hypoxic conditions, *Bmal1* is involved in the proliferation of superficial retinal vessels, while *Per2* contributes to the growth of deep retinal vessels [9]. These findings suggest that the *Bmal1* and *Per2* are closely associated with ectopic retinal angiogenesis. The deficiency of the clock gene *Bmal1* may facilitate the progression of diabetic retinopathy from NPDR to PDR, while its normal expression is likely to mitigate this pathological process. However, future in‐depth experimental studies are warranted to elucidate the precise mechanisms through which the clock gene *Bmal1* functions at different DR stages. Generally, the findings highlight the significance of the circadian clock system in maintaining vascular homeostasis, and suggest that the mammalian circadian clock is a pivotal regulator implicated in diverse retinopathies. As our comprehension of the critical role of circadian clock genes in disease pathophysiology continues to expand, future interventions targeting the pathways that govern mammalian circadian rhythms may offer a promising approach for developing innovative therapies against aging‐related disorders, retinopathies, neurodegenerative diseases, and tumorigenesis.

Overall, while direct evidence explicitly linking *Bmal1* to DR remains limited, its well‐documented role in modulating oxidative stress, inflammation, and vascular homeostasis highlights its potential as a pivotal therapeutic target. Additionally, the development of specific Bmal1 inhibitors and their integration with other therapeutic strategies may represent a promising avenue for the prevention and treatment of DR. Future research should prioritize unraveling the underlying mechanisms and performing rigorous clinical validation to fully exploit the therapeutic potential of *Bmal1*.

## 5 Crosstalk Between Bmal1 and Other Retinal Diseases

### 5.1 Role of Bmal1 in Glaucoma

Glaucoma, characterized by a group of chronically progressive disorders of the optic nerve, acts as the leading cause of irreversible blindness worldwide [104]. RGCs damage caused by elevated intraocular pressure (IOP) is a major etiologic factor for glaucoma [44]. Numerous studies have demonstrated that IOP exhibits an inherent circadian rhythm regulated by the light/dark cycle; interestingly, this IOP circadian rhythm is modulated by a combination of central and peripheral clock genes [105]. The circadian rhythm of IOP has a sinusoidal pattern which shows trough during subjective daytime and peak during subjective nighttime [106]. The central circadian clock genes of the SCN are subjected to the glucocorticoid‐mediated regulation. The *Bmal1* gene, whether expressing in SCN neurons or the paraventricular nucleus neurons, commit to controlling the daily rhythms of glucocorticoid release [107]. Glucocorticoids bind to and activate glucocorticoid receptors (GRs), enabling them to combine with response elements in DNA that are present at the promoter regions of clock genes such as *Per1* and *Per2* [108]. Extensive evidences have indicated that the disruption of either impaired central circadian clocks in SCN or glucocorticoid signaling removal results in the abrogation of circadian rhythm in IOP [109]. On the contrary, other components of the circadian clock genes such as CLOCK and CRY can inhibit the activation of GRs through interacting with them [110]. In the iris‐ciliary complex of mice, *Bmal1* gene expression is negatively correlated with IOP, but *Cry1, Cry2, Per1*, and *Per2* are positive correlated with IOP. Furthermore, it is worth noting that the alteration in *Bmal1*, *Cry*, and *Per* expression occurred prior to the change in IOP, suggesting that circadian clock genes may regulate the diurnal variation in IOP [111]. However, a separate study demonstrates that the IOP rhythm in ciliary epithelium‐specific *Bmal1* knockout mice remains intact, suggesting that the regulation of IOP rhythm may not be governed by the ciliary clock [112]. Hence, it is highly possible that the regulation of IOP rhythms is directly mediated by glucocorticoids rather than being governed by the circadian clock of the ciliary body. Whether peripheral clock genes are involved in the production of atrial water remains to be investigated. It is imperative to acknowledge that the central clock genes exert a substantial influence on the synthesis of aqueous humor. Although direct evidence is still limited, the regulatory roles of *Bmal1* in IOP, oxidative stress, and neuroinflammation highlight its potential as a promising therapeutic target for glaucoma. Integrating circadian biology with chronotherapeutics may provide novel strategies to preserve vision in this neurodegenerative condition.

### 5.2 Role of Bmal1 in Refractive Development and Myopia

Myopia is the most prevalent consequence of refractive error [113]. Myopia can cause various retinopathies, cataracts, and glaucoma, all of which are extremely blinding [114, 115, 116]. Recently, an increasing body of evidence has established a correlation between the circadian rhythms of ocular activity and refractive development. A myopia mouse model was generated by disrupting the circadian clock gene *Bmal1*, as embodied by a significant increase in both axial length and vitreous chamber depth of *Bmal1* knockout mice [117]. Mice with *Bmal1* defects also demonstrate a noticeable reduction in the amplitude of both scotopic and photopic full‐field ERG examinations, specifically in the b‐wave response. As it stands, circadian rhythms are associated with ocular growth and the development of refractive error and could offer a novel avenue for investigating the pathogenesis of myopia [113]. On the basis of these experimental results, it is reasonable to speculate that there may be a potential correlation between refractive development and the retinal circadian clock. Refractive dysgenesis or myopia in mammals, particularly humans, necessitates future research to substantiate the role of *Bmal1*.

### 5.3 Role of Bmal1 in Age‐Related Macular Degeneration

Age‐related macular degeneration (AMD), characterized by accumulation of extracellular deposits (namely drusen), progressive degeneration of photoreceptors and adjacent tissue, is a debilitating ocular disease that leads to severe visual impairment and legal blindness [118]. The relationship between AMD and circadian rhythms remains incompletely elucidated; nevertheless, there are several compelling reasons to posit a connection between them. RPE cells degeneration resulting from circadian rhythm dysregulation may be implicated in the progression of AMD. In the retina, RPE cells phagocytose photoreceptor outer segments (POS) to maintain photoreceptor function [39]. Phagocytosis of POS exhibits a circadian rhythm, with its peak occurring within 1–2 h of after light exposure [119]. Deletion of *Bmal1* from the RPE abolishes the daily peak of phagocytosis [26]. Another study demonstrates that the senescent RPE cells exhibit an upregulated phagocytic activity, a downregulated level of *Bmal1* mRNA, and an elevated level of *Rev‐erbα* mRNA [40]. Consequently, it is postulated that the dysregulation of circadian clock leading to enhanced RPE phagocytic activity may detrimentally impact retinal function in association with AMD [40]. Furthermore, claudin‐5, which is under regulation of the BMAL1 protein, exhibits the highest level of enrichment among a series of up to 30 interacting proteins within endothelial cells [120]. When the expression of claudin‐5 is persistently suppressed, it will induce significant atrophy in RPE cells. In the retina, iBRB plays an important role in maintaining retinal homeostasis [121]. Knockout of *Bmal1* in endothelial cells impedes claudin‐5 circulation in vivo and diminishes the levels of claudin‐5. The dysregulated circulation of claudin‐5 disturbs the iBRB's function in regulating substance exchange, thereby increasing the daily phagocytic load of RPE cells and leading to RPE atrophy and drusen deposition [122]. These findings suggest a critical role of an inner retina–derived factor in the early pathophysiological changes associated with AMD [122]. Therefore, restoring the integrity of the iBRB could potentially serve as a promising therapeutic strategy for preventing and treating geographic atrophy (GA) secondary to dry AMD. The maintenance of stable expression of clock genes and five proteins in the retina may be effective in the prevention or halting of the progression of AMD.

Additionally, the activation of Vegf has been implicated in the pathogenesis of choroidal neovascularization (CNV) [123, 124]. In zebrafish, researchers have discovered that the CLOCK:BMAL1 heterodimer can enhance the expression of *Vegf* via targeting the promoter region of *Vegf* gene. Conversely, deletion of these E‐boxes in the promoter region results in the inactivation of *Vegf* gene promoter activity [125]. In light of the highly conserved nature of the clock genes, these results can be reasonably extrapolated to mammals. The results of another study demonstrate that stable silencing of BMAL1 can mitigate the expression of VEGF [126]. Furthermore, it has been observed that the *Bmal1* gene exerts an indirect regulatory effect on VEGF expression through modulation of the WNT/β‐catenin pathway [123, 124]. Dysregulation of circadian rhythm may result in the activation of the WNT/β‐catenin pathway, which subsequently contributes to wet AMD [127, 128]. Specifically, BMAL1 regulates multiple genes involved in WNT signaling [128]. Moreover, *Bmal1* is implicated in both transcription and degradation of β‐catenin [129]. The WNT/β‐catenin pathway directly upregulates the VEGF expression in wet AMD [130]. Conversely, the WNT/β‐catenin pathway activates the PI3K/Akt pathway through inhibiting GSK‐3β [124]. The activition of PI3K/Akt pathway has been demonstrated in wet AMD, leading to subsequent upregulation of *Vegf* expression [131, 132, 133]. Therefore, it is reasonable to hypothesize that the dysregulation of *Bmal1* may be associated with the increased in VEGF level in wet AMD. Retinoic Acid 6 (STRA6) is a multi‐transmembrane protein that serves as a specific membrane receptor for retinol binding protein (RBP) and demonstrates high affinity in binding to RBP [134, 135]. The disruption of circadian rhythms has been shown to facilitate the development of CNV. Studies have shown that patients with AMD, exhibit significantly elevated levels of STRA6 expression compared with healthy controls [8]. Furthermore, STRA6 is found to be enriched in pathways associated with angiogenesis. STRA6 expression is under the control of the promoters CLOCK and BMAL1, which regulates the circadian cycle. In vivo experiments demonstrated that STRA6 knockdown reversed the CNV formation induced by circadian rhythm disruption. STRA6 knockdown has been shown to significantly impair the proliferation, migration, and VEGF secretion of RPE cells in vitro. Additionally, the STRA6‐induced CNV formation in asynchronous RPE cells is found to be mediated through the activation of the JAK2/STAT3/VEGFA pathway. These studies elucidate the importance of circadian rhythms in AMD‐associated neovascularization, providing new pathways and targets for anti‐VEGF therapy.

## 6 Therapeutic Potentials of Bmal1

**FIGURE 4:**
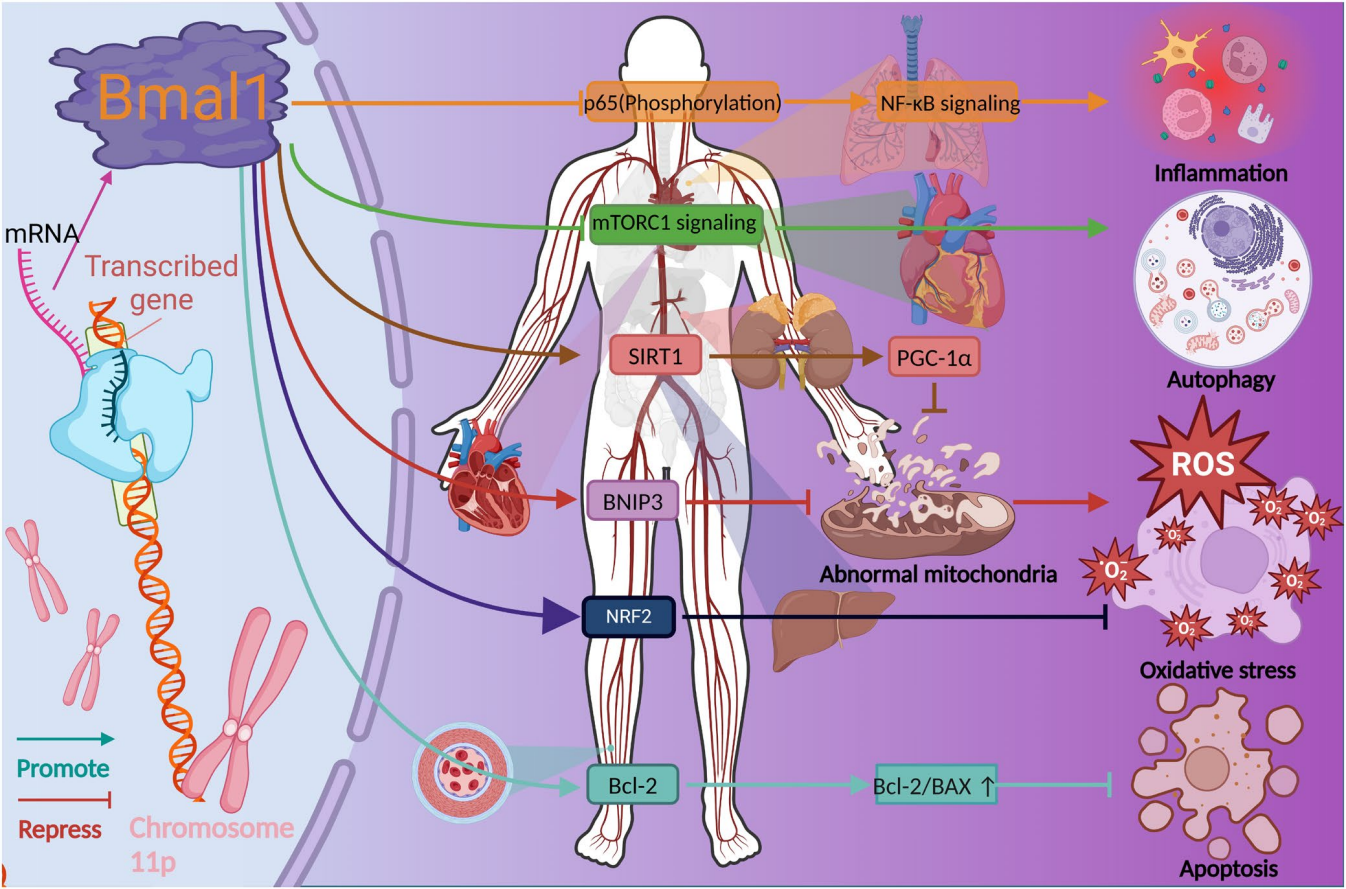
The molecular mechanism of *Bmal1*, which is involved in inflammation, autophagy, oxidative stress, and apoptosis.

Currently, the targeting of the *Bmal1* gene for therapeutic aim is a burgeoning area of research (Figure 4). In patients with Parkinson's disease (PD), the baseline levels of relative expression of the *Bmal1* gene at approximately 9:00 am are found to be 57% lower than those of the *Per1* gene in peripheral blood [136, 137]. In contrast, the relative expression level of the *Bmal1* gene in the peripheral blood of healthy individuals is approximately 30% lower than that of the *Per1* gene. Another study also shows that the PER1:BMAL1 ratio undergoes significant alterations in PD patient, and the researchers examine the melatonin mediated effects on the expression of clock genes in the peripheral blood of PD patients [138]. Through a 3 month administration of melatonin, the BMAL1 level is observed to increase in the morning, showing a pattern similar to that of the placebo group. However, melatonin demonstrates greater efficiency in enhancing the BMAL1 level compared with the placebo. Hence, the stability of the circadian rhythm cycle is, to some extent, correlated with the prognosis of PD; however, further research is warranted to verify this association. Researchers have documented acute postprandial effects of meal timing on the expression level of clock and clock‐controlled genes in both healthy individuals and those with type 2 diabetes. Specifically, omitting breakfast can disrupt acutely the circadian rhythms in both healthy individuals and patients with type 2 diabetes [139]. Breakfast skipping and/or eating during hours typically designated for sleep can, disrupt the expression of clock genes and are associated with weight gain as well as an increased risk of diabetes [140, 141]. Furthermore, breakfast skipping in healthy individuals has been shown to alter the expression patterns of clock genes. After a test day with only lunch provided, *Sirt1*, *Clock*, *Bmal1*, *Per2*, *Ampk*, and *Ror*α genes are up‐regulated, *Per1* is down‐regulated, and *Cry1* expression remains unchanged. These alterations disrupt the normal cyclic expression patterns of these genes. Consequently, modulating clock genes could potentially serve as a therapeutic strategy for diabetes and DR in future work.

However, the precise impact of the *Bmal1* gene on retinopathy remains elusive. Therefore, we intend to elucidate several mechanisms underlying retinopathy amenable to *Bmal1* gene regulation. To our knowledge, the *Bmal1* gene plays a crucial role in maintaining mitochondrial homeostasis, as evidenced by aberrant mitochondrial morphology and significant functional impairment observed in *Bmal1* knockout cardiomyocytes [142]. In diabetic cardiomyopathy, *Bmal1* overexpression can alleviate Bcl2/IP3R‐mediated mitochondrial Ca^2+^ overload to decrease cell apoptosis [143]. Peroxisome proliferator‐activated receptor gamma coactivator 1 alpha (PGC‐1α) is a crucial regulator of mitochondrial biogenesis and participates in promoting mitochondrial protein synthesis and functional mitochondrial regeneration. BMAL1 binds to E‐box elements to upregulate the transcription of silent information regulator 1 (SIRT1) [144]. SIRT1 can promote mitochondrial biogenesis via the activation of PGC‐1α. In terms of inflammatory effects, BMAL1 primarily exhibits anti‐inflammatory effects, whereas its heterodimeric partner, CLOCK, can promote inflammatory responses. BMAL1 indirectly inhibits the NF‐κB signaling pathway, which activates the secretion of various pro‐inflammatory cytokines in the endothelial cells of lining the arteries, such as IL‐6, IL‐1β and TNF‐α, and directly controls nuclear factor erythroid 2‐related factor 2 (NRF2) transcriptional activity in macrophages [93, 145]. These key factors, which are regulated by *Bmal1*, play a crucial role in retinal diseases. In this context, targeting the *Bmal1* gene could act as a viable approach to address retinal diseases. For instance, simvastatin plays a protective role in retinal neuron degeneration following ischemia by improving the Bcl‐2/BAX ratio [146]. Researchers propose that regulating SIRT1 expression through targeting the *Bmal1* gene may be effective to mitigate the progression of AMD. Moreover, studies have shown that the enhanced SIRT1 activity elicited by melatonin results in a decrease in inflammatory response and oxidative stress in DR [147]. NRF2, a crucial transcription factor for antioxidants, is significantly linked with age‐related ailments like AMD and DR. Additionally, BMAL1 exerts regulatory control over Nrf2 mRNA expression through directly binding to its promoter via E‐box motifs [148]. Importantly, accumulating evidence suggests that *Bmal1* plays a crucial role in various pathological processes of retinal diseases, including the regulation of mitochondrial homeostasis and energy metabolism. However, disturbances of these functions would impair eye homeostasis. This further emphasizes the crucial importance of investigating the intricate correlation between *Bmal1* and retinopathy to achieve a more profound understanding of the circadian‐related disorders. Recently, Core Circadian Modulator (CCM) has demonstrated its ability to specifically target the PASB domain pocket of BMAL1, inducing protein conformational changes, thereby regulating circadian rhythms and immune‐related gene expression. The mechanism of action of CCM differs from that of other regulators, as its modulatory effect on BMAL1 is gene‐selective. These findings not only provide novel insights into the mechanisms of circadian rhythm regulation, but also reveal that BMAL1 can achieve functional modulation through direct compound binding [149]. Notably, *Bmal1* also plays a critical role in orchestrating both innate and adaptive immune responses, influencing diverse aspects of immune cell biology such as development, differentiation, migration, homing, metabolism, and effector function [10, 11, 12]. Intestinal flora imbalance, which disrupts the microecological equilibrium, can result in immune and metabolic dysfunction of the host, consequently influencing the progression of numerous diseases. Dysregulation of the gut microbiota has been implicated in the onset and progression of AMD via mechanisms such as lipid abnormalities, oxidative stress, and cellular crosstalk between microglia and other cell types, thereby promoting immune‐mediated inflammatory responses within the eye [150]. Consequently, achieving a deeper understanding of the association between the clock gene *Bmal1*, gut microbiome, and the immune microenvironment holds considerable potential for the treatment of retinopathies. On the basis of these experimental data, it is plausible to hypothesize that therapeutic targeting of *Bmal1* may yield beneficial effects in the treatment of retinal diseases through the aforementioned mechanisms or pathways.

## 7 Current Challenges and Future Perspectives

Despite the rapid advancements in understanding the clock gene *Bmal1* in the retina, its clinical application for retinal diseases remains to be explored. To promote its future clinical translation, it is necessary to address several key challenges: (1) Complexity and multi‐factoriality: The correlation between the circadian rhythm and retinopathy is inherently complex, as the pathologic processes are influenced by multiple interconnected factors. The molecular network through which circadian rhythm regulates physiological functions is characterized by a high degree of coordination. However, the specificity of the circadian rhythm in different tissues, in addition to the effects of external factors such as diet and light exposure, contributes to the complexity of the system, thus making comprehensive analyses challenging. This underscores the necessity for developing comprehensive methodologies that consider the interconnected nature of these factors, thereby facilitating a more nuanced and universally applicable understanding of the circadian mechanisms underlying retinal diseases. (2) Limited clinical evidences: Although there are experiments exploring the therapeutic potential of the clock gene *Bmal1* in retinal diseases, the lack of clinical trials remains a substantial barrier. It is essential to recognize that different retinal diseases exhibit distinct characteristics, thus requiring a deeper understanding of the *Bmal1* mediated effects in the specific contexts. This not only necessitates intensified research into the role of the clock gene *Bmal1* in retinal diseases but also demands exploration of the interactions among other genes, proteins, and signaling pathways implicated in the pathological process. These findings could potentially pave the way for targeted therapeutic interventions, thereby contributing to the development of novel therapeutic strategies for various retinopathies.

## 8 Outlook

Retina possesses a sophisticated circadian system in which individual cells exhibit autonomous oscillations. The circadian oscillators are synchronized to regulate the rhythms of physiological function. Circadian rhythms in ocular biology constitute a pivotal domain, given that disrupted circadian function can precipitate eye diseases. Accumulated data have substantiated the pivotal role of *Bmal1* in retinal physiology, encompassing visual signaling pathways, vitality of retinal neurons, and the pattern of neurotransmitter release. Ongoing investigations across diverse animal models indicate that *Bmal1* is implicated in the pathogenesis of retinopathy, highlighting that targeting *Bmal1* gene‐related loci or *Bmal1* expression itself could be pertinent to treating retinal diseases. Current evidence suggests that *Bmal1* exhibits intricate interactions with the neuronal activity of SCN. Additionally, it exerts its influence on circadian rhythmicity beyond various retinal cell types, including photoreceptors, RGCs, and astrocytes, encompassing crucial molecular components such as dopamine, melatonin, and REV‐ERBα, among others. The coordination and maintenance of retinal circadian functions and visual circuitry involve the integration of these factors within a complex network.

The retinal circadian rhythm plays a crucial role in maintaining normal retinal physiology, ensuring the structural integrity and functionality of retinal circuits. Disruption of the circadian rhythm has been demonstrated to cause alterations in the fate of *Bmal1*, which is associated with an elevated risk of retinal diseases, such as DR, AMD, and glaucoma. *Bmal1* has emerged as a promising target for the treatment and prevention of retinal diseases. A multitude of recent studies have focused on the *Bmal1‐mediated* effects on retinal cells, such as the photoreceptors and glial cells [7, 151]. The disruption of the biological clock accelerates photoreceptor degeneration, thereby establishing a significant connection between circadian rhythm disturbances and retinal degeneration. Moreover, the circadian clock within retinal Müller glial cells is crucial for neuronal survival, vascular integrity maintenance, and proper retinal function. Another pioneering research project has also shown that *Bmal1* can affect the prognosis of retinopathy through modulating specific pathophysiological processes. This has raised the possibility of translating this knowledge into clinical trials. For instance, the dysregulation of claudin‐5 expression in the inner retinal layer disrupts the integrity of the blood‐retinal barrier, contributing to the atrophy of RPE cells, which process plays a critical role in the early development of AMD [122]. Future research is required to identify effective *Bmal1* regulators and to develop effective interventions for a range of retinal diseases.

## Author Contributions

Conceptualization, Y.C. and Y.T.; Writing – original draft, Y.C. and H.L.; Writing – review and editing, Y.T.; Methodology, J.W. and Y.T.; Formal analysis, W.F., H.W., C.Q., and N.P.; Supervision, Y.T. All authors have read and agreed to the published version of the manuscript.

## Conflicts of Interest

The authors declare no conflicts of interest.

## Data Availability

Data will be made available on request.
